# Supplementary material for: Ecosystem size-induced environmental fluctuations affect the temporal dynamics of community assembly mechanisms
Source: ISME J. 2022 Aug 18;16(12):2635–43. doi: 10.1038/s41396-022-01286-9 (PMC9666552; doi:10.1038/s41396-022-01286-9)
Supplement: Supplementary file 1 — Supplementary Information [file 41396_2022_1286_MOESM1_ESM.pdf]

<u>Supplementary Information for:</u> Ecosystem size-induced environmental fluctuations affect the temporal dynamics of community assembly mechanisms

Raven L. Bier*, Máté Vass, Anna J. Székely, Silke Langenheder

*Corresponding author contact information:

Raven Bier

Savannah River Ecology Laboratory

University of Georgia

PO Drawer E

Aiken SC 29802 USA

Phone: +1 803-725-9726

Email: rbier@srel.uga.edu

This file includes:

Supplementary Methods and References

Supplementary Tables S2-S7

Supplementary Figures S1-S9

Supplementary Table S1 is supplied as a separate .xlxs file

Supplementary Methods

Experimental set-up and monitoring equipment

The initial Lake Erken water measurements were as follows: 17.94 mg L^{-1} total organic carbon (TOC), 0.671 mg L^{-1} total nitrogen (TN), 0.016 mg L^{-1} total phosphorus (TP), 25.86 relative fluorescence units (rfu) chlorophyll-*a* (chl-*a*), and 0.224 µm L^{-1} colored fraction of dissolved organic matter (CDOM). The upper limit of salinity that was created for the most saline mesocosm in the gradient of 16 mesocosms was 6 ‰ and corresponds to Baltic Sea salinity at Lake Erken latitude (1). Lake Erken is < 20 km from the coast and likely to have Baltic Sea bacteria in its water and sediment seed banks. This also entrains a likelihood that Baltic Sea bacteria dispersed through air and precipitation into the mesocosms. The mesocosm at the least saline end of the gradient had no salt added to it.

Water sample monitoring was conducted for conductivity and temperature (Cond 3210 conductivity meter Xylem Analytics Germany Sales GmbH & Co. KG, Weilheim, Germany), and depth integrated pH (micropH 2001 Crison Instruments, S.A., Alella, Spain), chlorophyll-*a*, and CDOM fluorescence (Aqua Fluor Handheld Fluorometer/Turbidimeter Turner Designs, San Jose, CA, USA at 395 nm and 350 nm, respectively). These and other environmental data including mesocosm depth on each sampling date are available in the Swedish institutional repository, DiVA, (diva-portal.org) with the following accession number: diva2:1210995.

Polymerase Chain Reaction (PCR) Conditions

Extracted total RNA was treated with DNase I (Invitrogen, Carlsbad, CA, USA), and a subsample was checked for residual DNA using a 35-cycle PCR amplification program before a fresh aliquot was reverse transcribed. The conditions for this DNA-check PCR program were initial denaturation at 98 °C for 30 seconds, 35 cycles of 98 °C denaturation for 10 seconds, 48

°C annealing for 30 seconds, and 72 °C extension for 30 seconds, and after the end of the 35 cycles, a 2 minute extension at 72 °C.

The conditions for the first PCR program to amplify cDNA, which used Illumina adaptor attached primers, were as follows: initial denaturation at 98 °C for 3 minutes, 20 cycles of 98 °C denaturation for 10 seconds, 48 °C annealing for 30 seconds, and 72 °C extension for 30 seconds, and after the end of the 20 cycles, a 2 minute extension at 72 °C. The conditions for the second PCR program, which attached standard Illumina handles and index primers, were as follows: initial denaturation at 98 °C for 30 seconds, 15 cycles of 98 °C denaturation for 10 seconds, 66 °C annealing for 30 seconds, and 72 °C extension for 30 seconds, and after the end of the 15 cycles, a 2 minute extension at 72 °C. For both programs, samples were held at 6 °C once the program was completed.

Additional data processing details

Sequences were trimmed to 280 bp and 200 bp for forward and reverse reads, respectively, and merged with at most two expected errors. Following dereplication and chimeric sequence removal from the Amplicon Sequence Variants (ASVs), the SILVA v. 138.1 reference database (2) (August 2020) was used to assign taxonomy at 99 % threshold. ASVs not identified as Bacteria were removed. Forty ASVs identified as extraction or PCR contaminants using R package “decontam” v 1.8.0 ref. (3) with default settings using the prevalence-based approach were removed from 11 samples leaving 12 068 ASVs.

Network analysis details

For equal time windows between samplings (8-days), samples from day 2 and 4 were removed. Samples from day 64 were also removed due to the low sequence retention (Table S2). The 50 most abundant ASVs from each size category that were used comprised, on average, 70

% (range 53–76 %), 72 % (range 59–80 %), and 63 % (range 47–72 %) of the relative bacterial abundance in small, medium, and large mesocosms, respectively. 'Simple' method (simple average method) was used to summarize replicate data, and p-values for pairwise LS correlations were determined using the 'mixed' approach (4). LS values were considered statistically significant if $p \leq 0.01$ and Q (the false discovery rate) ≤ 0.01.

References

1. DeFaveri J, Jonsson PR, Merila J. Heterogeneous genomic differentiation in marine threespine sticklebacks: adaptation along an environmental gradient. Evolution. 2013;67(9):2530-46.
2. Quast C, Pruesse E, Yilmaz P, Gerken J, Schweer T, Yarza P, et al. The SILVA ribosomal RNA gene database project: improved data processing and web-based tools. Nucleic Acids Res. 2013;41:D590-6.
3. Davis NM, Proctor DM, Holmes SP, Relman DA, Callahan BJ. Simple statistical identification and removal of contaminant sequences in marker-gene and metagenomics data. Microbiome. 2018;6(1):226.
4. Xia LC, Ai D, Cram J, Fuhrman JA, Sun F. Efficient statistical significance approximation for local similarity analysis of high-throughput time series data. Bioinformatics. 2013;29(2):230-7.

Table S1. Statistics of sequences from DADA2 sequence processing. (See Excel file)

Table S2. Samples not meeting the 5028 sequence per sample evenness requirement for inclusion in beta diversity analysis.

Sample Day	Mesocosm ID	Total Sequences
D16	19	0
D56	13	5
D48	29	752
D24	2	1993
D64	33	2067
D64	48	2276
D64	45	2527
D64	31	2546
D64	6	2793
D64	12	2952
D64	37	3064
D64	39	3189
D64	17	3353
D64	44	3541
D64	Rain	3783
D64	36	3900
D64	5	3939
D64	40	4106
D56	Air	4137
D64	15	4161
D64	Air	4401
D64	27	4479
D64	47	4626
D32	1	4666
D64	43	4670
D64	16	4770
D64	35	4982
D64	11	4996

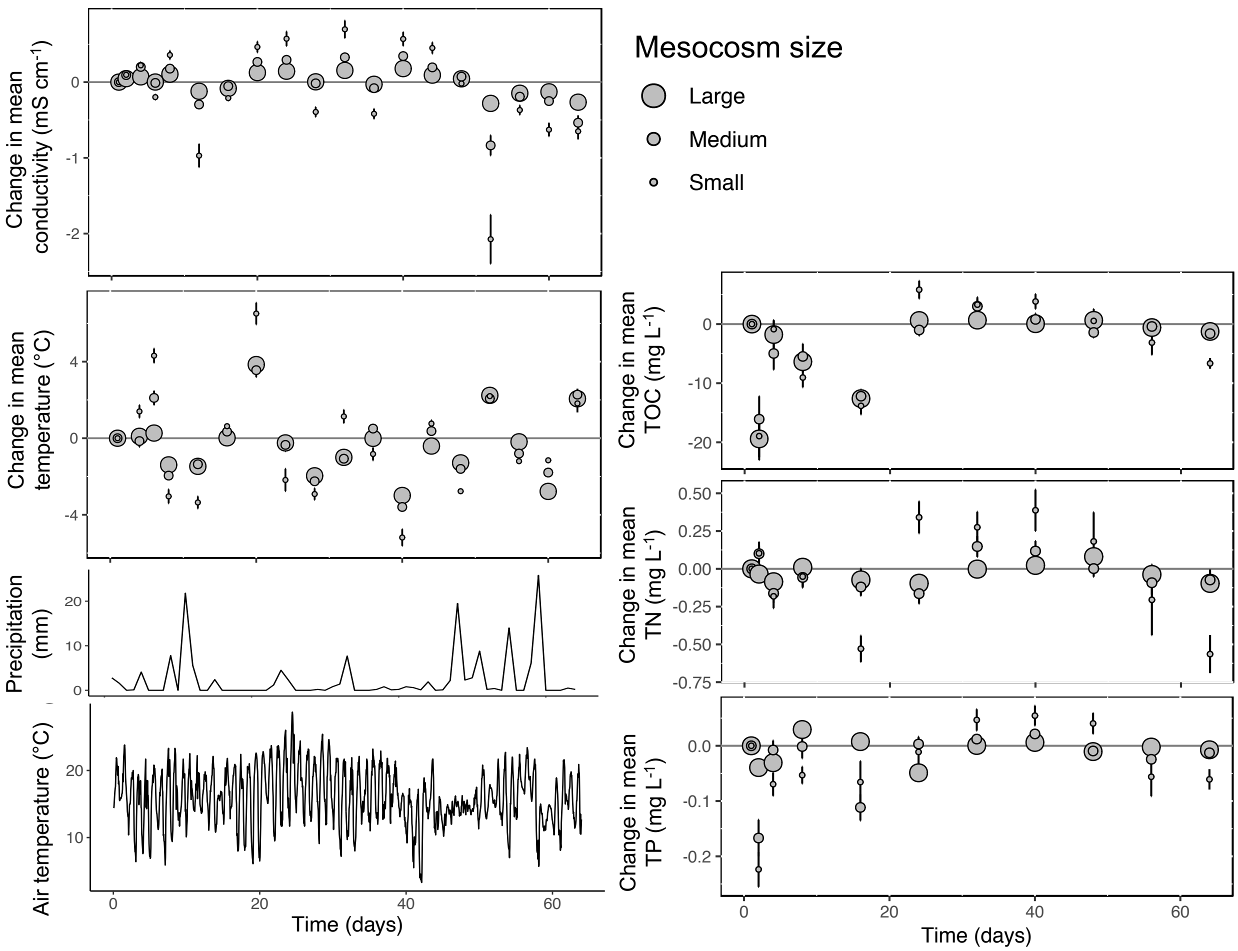

Figure S1. Change in mean conductivity, water temperature, and nutrients of experimental mesocosms from previous sampling day. Daily precipitation and air temperature were gathered from Svanberga station, Sweden, SMHI. Error bars represent standard error from the mean. (n = 16)

Table S3. Results from nonparametric tests with an ANOVA-type statistic for repeated measures to compare changes in environmental variables between mesocosm sizes.

Environmental Variable (Absolute Change)	Category	Term	Statistic	df	p-value
Conductivity (mS/cm)	Overall	Size	7.93	1.9	**5.15E-04**
		Day	86.52	3.4	**1.81E-62**
		Size:Day	5.15	5.5	**5.47E-05**
	Small vs Medium	Size	2.99	1.0	8.40E-02
	Small vs Large	Size	15.97	1.0	**6.44E-05**
	Medium vs Large	Size	5.50	1.0	**1.90E-02**
Temperature (°C)	Overall	Size	108.35	1.7	**1.64E-40**
		Day	91.84	4.7	**4.08E-91**
		Size:Day	11.75	6.4	**6.18E-14**
	Small vs Medium	Size	74.55	1.0	**5.91E-18**
	Small vs Large	Size	190.41	1.0	**2.59E-43**
	Medium vs Large	Size	33.15	1.0	**8.54E-09**
Chlorophyll-a (RFU)	Overall	Size	7.23	1.5	**2.05E-03**
		Day	19.94	10.3	**1.82E-38**
		Size:Day	2.66	16.2	**3.06E-04**
	Small vs Medium	Size	3.83	1.0	5.03E-02
	Small vs Large	Size	12.28	1.0	**4.58E-04**
	Medium vs Large	Size	4.66	1.0	**3.09E-02**
CDOM (mg/L)	Overall	Size	32.68	1.6	**2.91E-12**
		Day	26.69	10.7	**5.17E-55**
		Size:Day	6.27	16.7	**9.05E-15**
	Small vs Medium	Size	22.84	1.0	**1.76E-06**
	Small vs Large	Size	48.72	1.0	**2.95E-12**
	Medium vs Large	Size	14.59	1.0	**1.33E-04**
pH	Overall	Size	4.13	1.9	**1.86E-02**
		Day	0.59	4.4	6.88E-01
		Size:Day	3.34	8.0	**7.98E-04**
	Small vs Medium	Size	0.95	1.0	3.29E-01
	Small vs Large	Size	3.72	1.0	5.37E-02
	Medium vs Large	Size	6.39	1.0	**1.15E-02**
Cell Abundance (cells/mL)	Overall	Size	16.81	2.0	**5.67E-08**
		Day	26.37	10.1	**1.61E-51**
		Size:Day	3.69	16.5	**5.21E-07**
	Small vs Medium	Size	1.83	1.0	1.77E-01
	Small vs Large	Size	31.61	1.0	**1.89E-08**
	Medium vs Large	Size	18.75	1.0	**1.49E-05**
TOC (mg/L)	Overall	Size	16.76	1.9	**1.27E-07**
		Day	30.35	6.5	**1.26E-39**
		Size:Day	2.02	11.1	**2.23E-02**
	Small vs Medium	Size	4.87	1.0	**2.74E-02**
	Small vs Large	Size	32.34	1.0	**1.30E-08**
	Medium vs Large	Size	14.68	1.0	**1.27E-04**
TN (mg/L)	Overall	Size	41.10	1.9	**6.96E-18**
		Day	3.11	7.4	**2.31E-03**
		Size:Day	2.07	12.3	**1.48E-02**
	Small vs Medium	Size	18.84	1.0	**1.42E-05**
	Small vs Large	Size	87.81	1.0	**7.21E-21**
	Medium vs Large	Size	21.50	1.0	**3.54E-06**
TP (mg/L)	Overall	Size	24.25	1.8	**2.03E-10**
		Day	20.27	7.2	**3.10E-28**
		Size:Day	1.24	12.1	2.45E-01
	Small vs Medium	Size	10.53	1.0	**1.18E-03**
	Small vs Large	Size	66.49	1.0	**1.99E-15**
	Medium vs Large	Size	10.93	1.0	**9.46E-04**

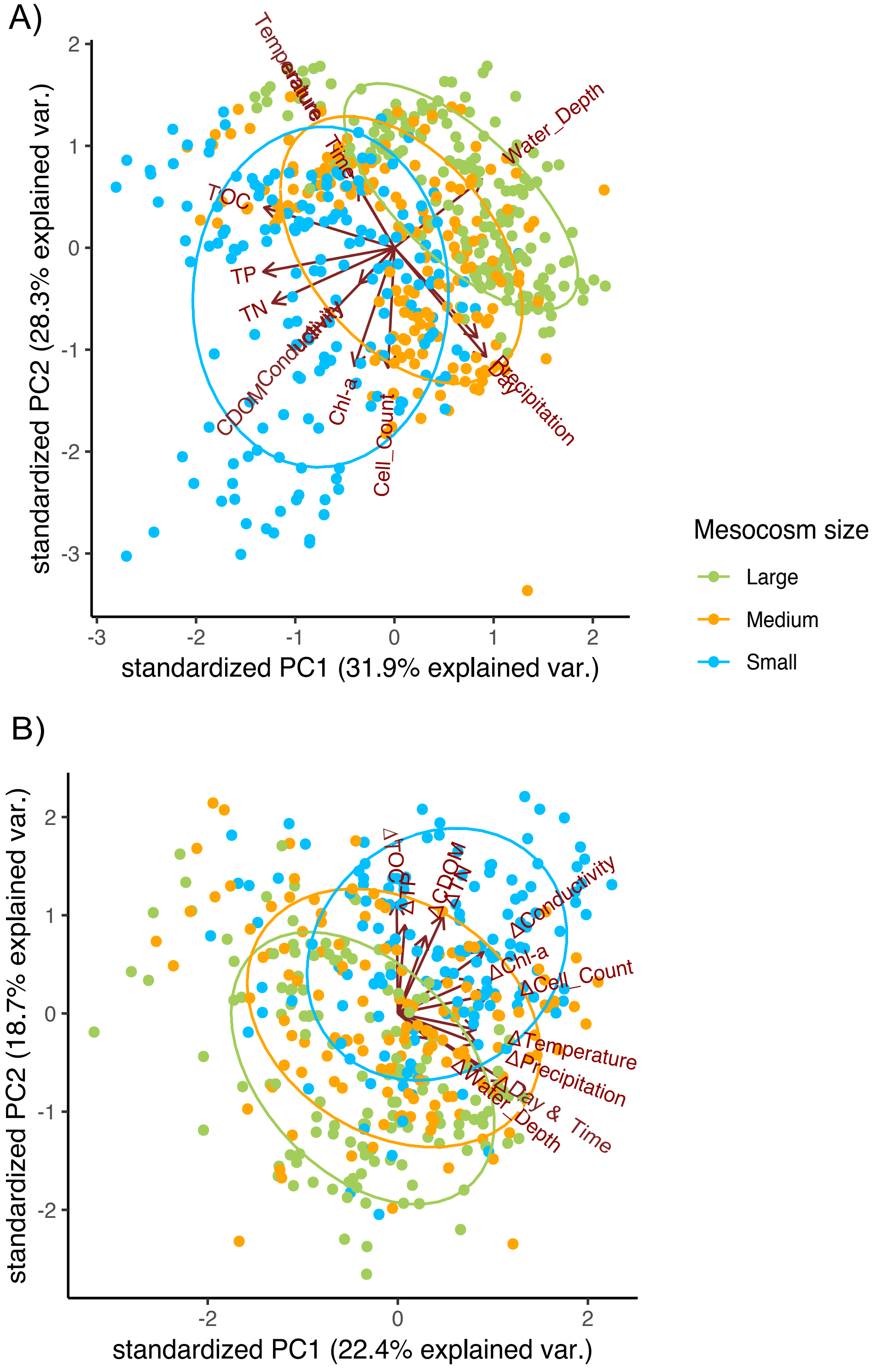

Figure S2. Principal components analysis plots of log transformed environmental variables using raw data (A) or absolute changes from sampling day-to-sampling day (B). Different colors show mesocosm size. Environmental vector correlations shown were significant at $p < 0.05$.

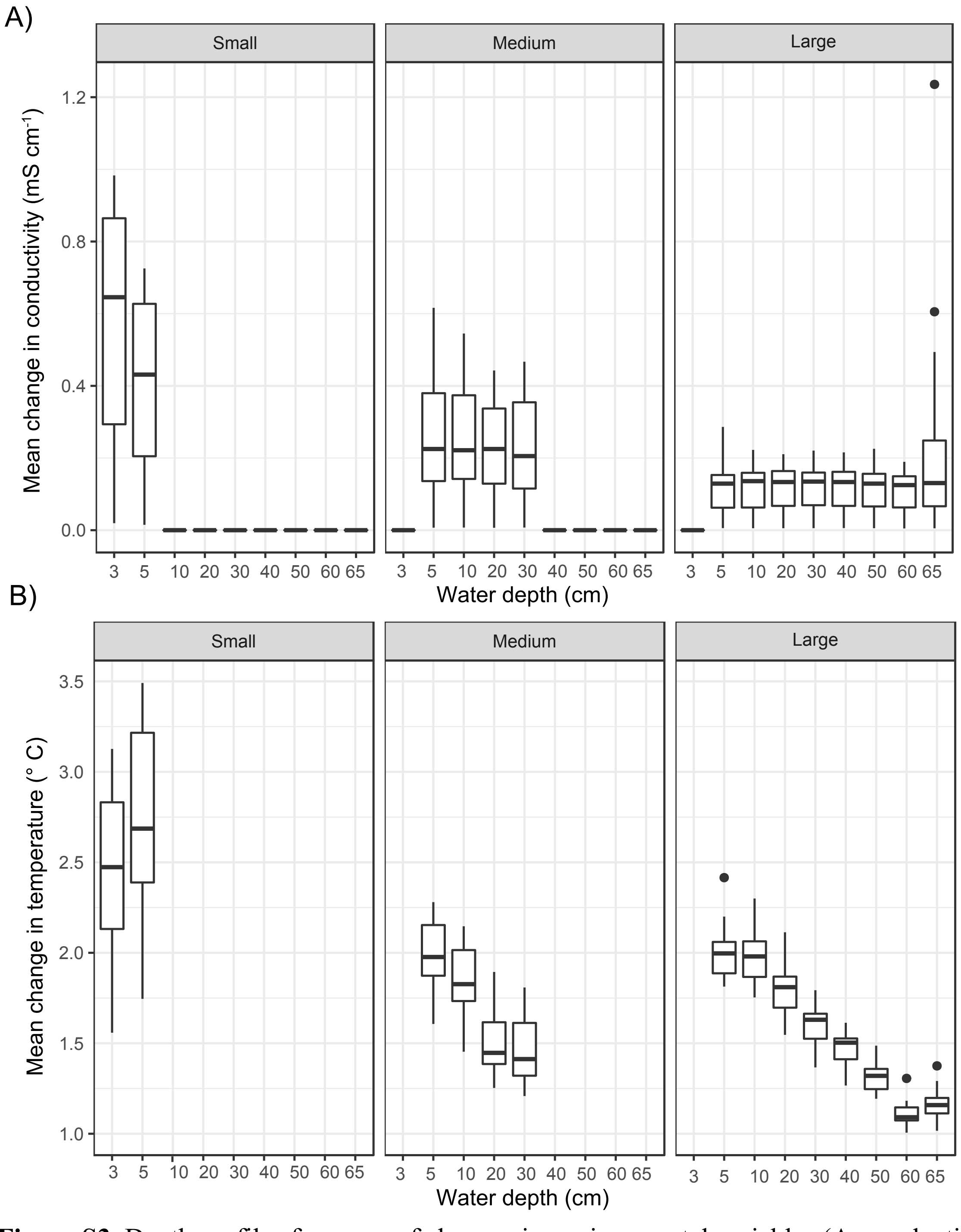

Figure S3. Depth profiles for mean of changes in environmental variables (A: conductivity, B: temperature) over experiment duration (64 days) in mesocosms of different size. (n = 16)

A)

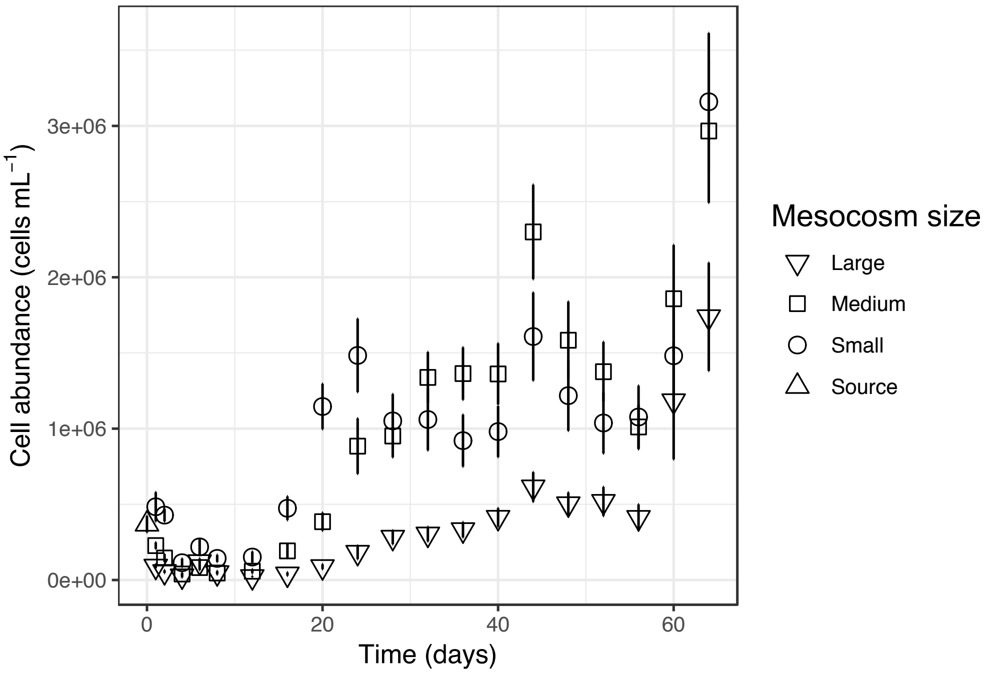

B)

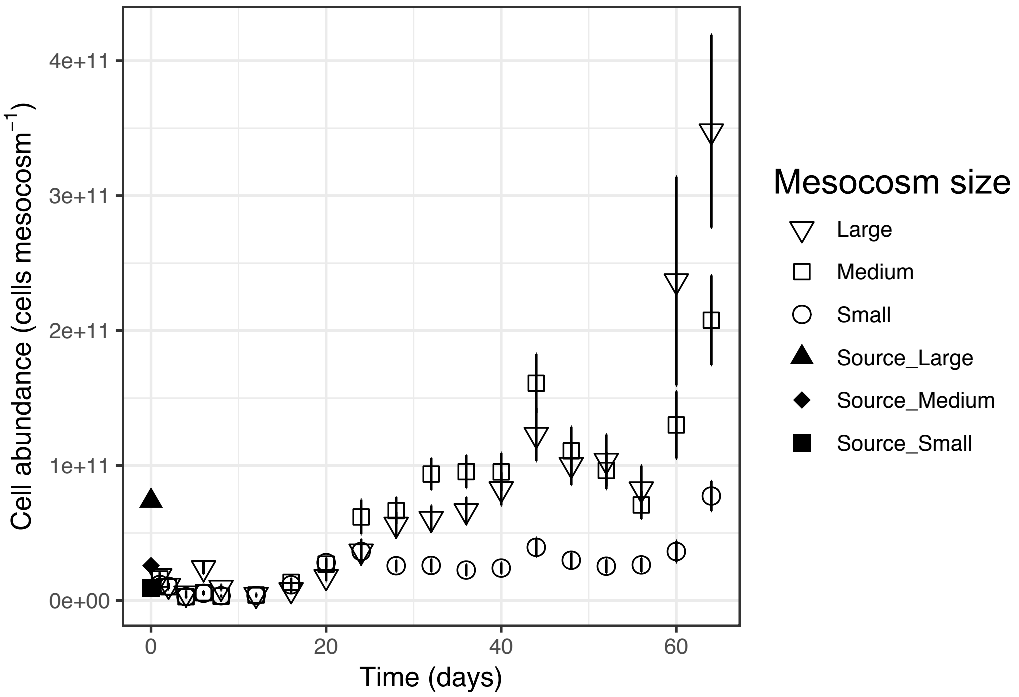

Figure S4. Cell abundance as mean cells mL^{-1} (A) and total cells per entire mesocosm (B). Source is cell abundance given from lake source at Day 0 and Source_*size* is cell abundance from each mesocosm size on Day 0. Error bars are standard error. (n = 16)

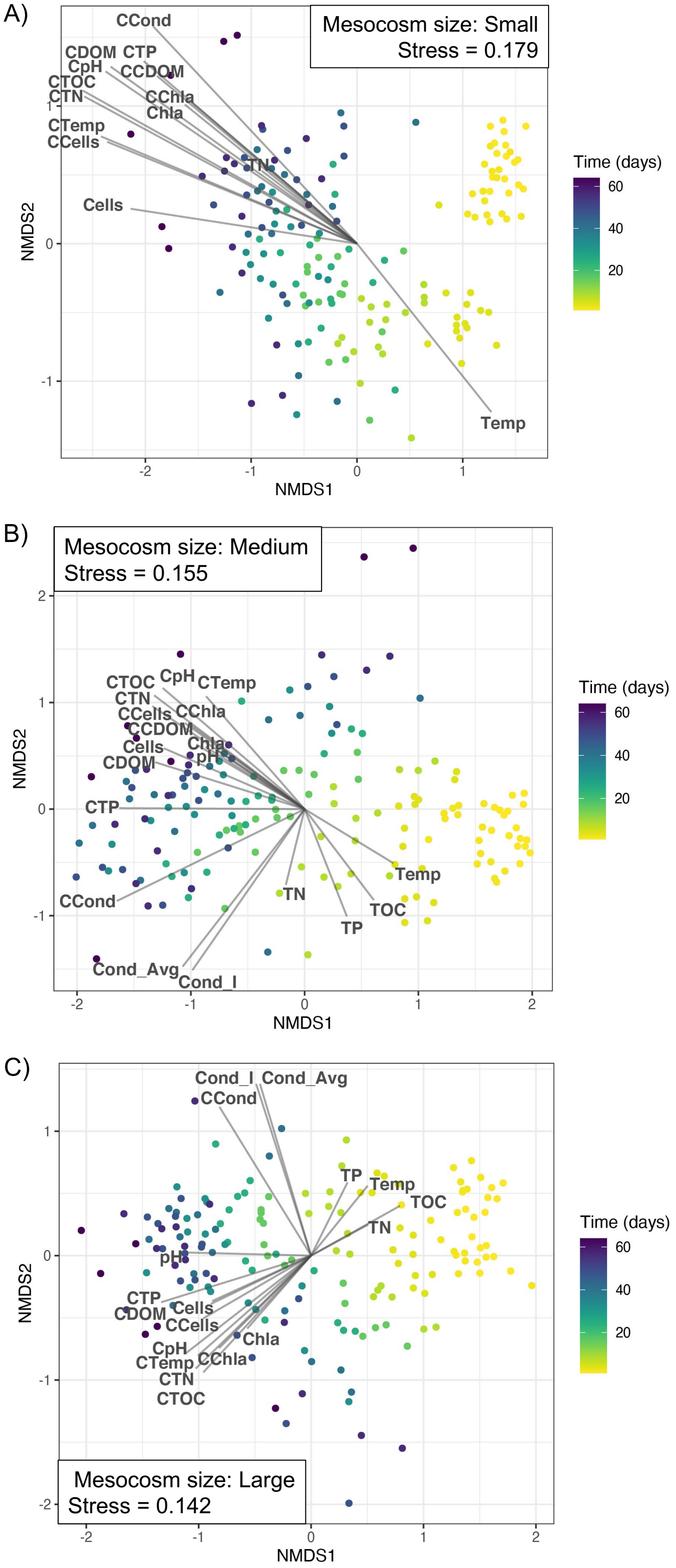
A)
Mesocosm size: Small
Stress = 0.179
CCond
CDOM CTP
CpH
CCDOM
CTOC
CTN
CChla
Chla
CTemp
CCells
TN
Cells
Temp
NMDS1
NMDS2
Time (days)
B)
Mesocosm size: Medium
Stress = 0.155
CTOC
CpH
CTemp
CTN
CCells
CChla
CCDOM
Chla
Cells
pH
CDOM
CTP
CCond
Cond_Avg
Cond_I
TN
TP
TOC
Temp
C)
Cond_I
Cond_Avg
CCond
TP
Temp
TOC
TN
pH
CTP
CDOM
Cells
CCells
Chla
CpH
CTemp
CChla
CTN
CTOC
Mesocosm size: Large
Stress = 0.142

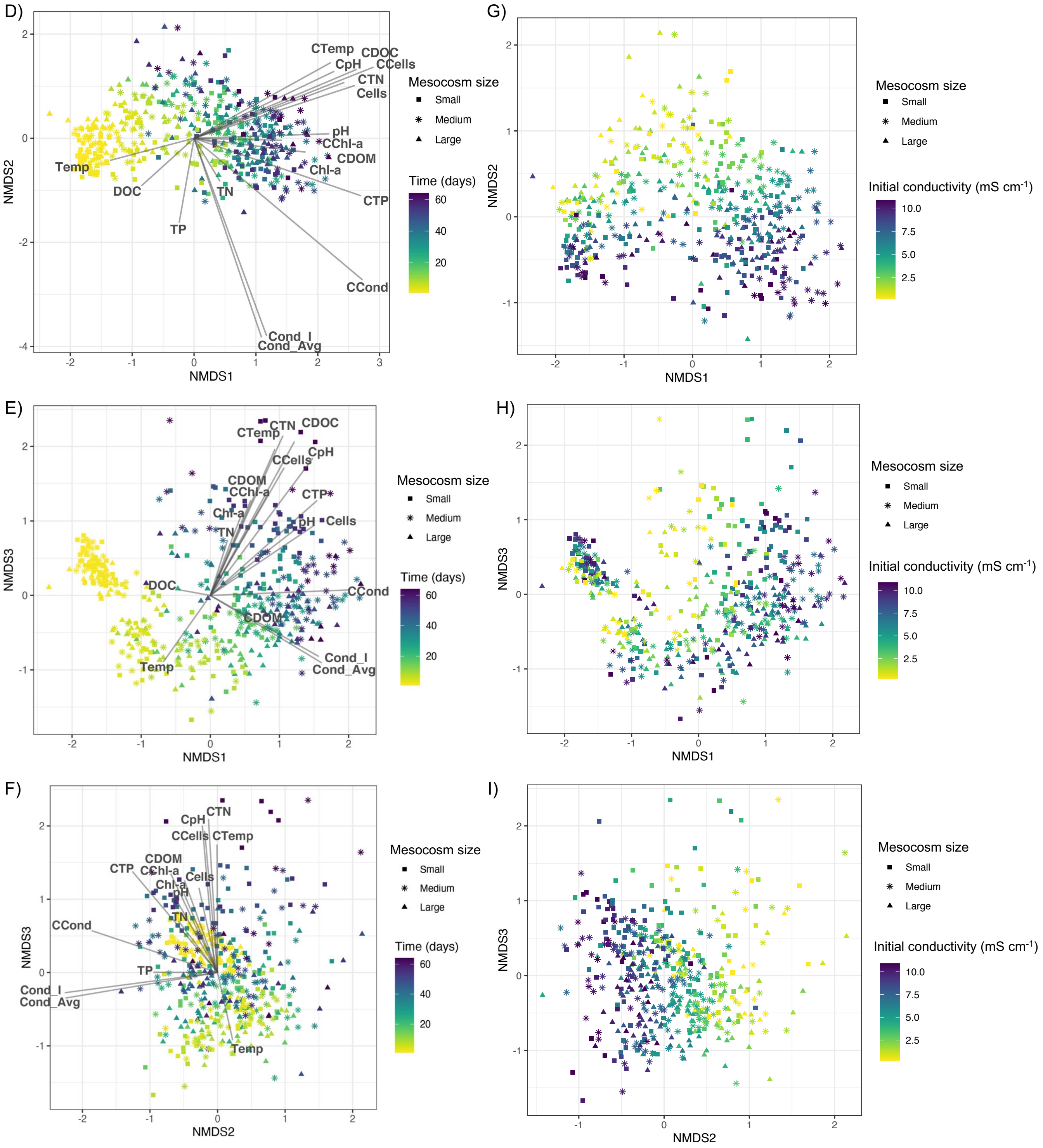

Figure S5. Nonmetric multidimensional scaling graphs of Bray-Curtis dissimilarity matrices for bacterial communities in small (A), medium (B), and large (C) mesocosm sizes and in all mesocosm sizes with environmental vectors and points colored by time (D-F) or by initial conductivity (G-I) shown with different NMDS axes. 'C' in environmental vector names indicates cumulative change. When applicable, environmental vectors were depth averaged values. Cond_I signifies initial mesocosm conductivity from conductivity gradient. NMDS plots

repeated to show all combinations of two of the three axes. All environmental vectors $p < 0.05$. NMDS stress = 0.128.

A) Small mesocosm

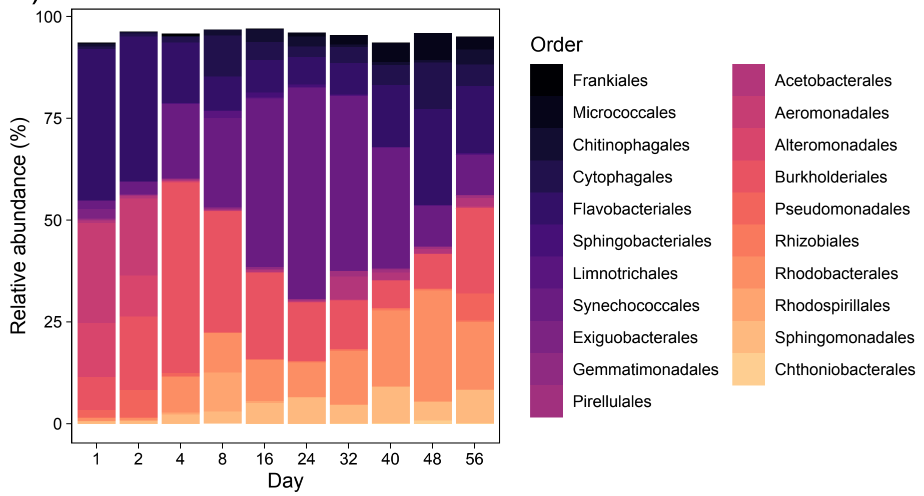

B) Medium mesocosm

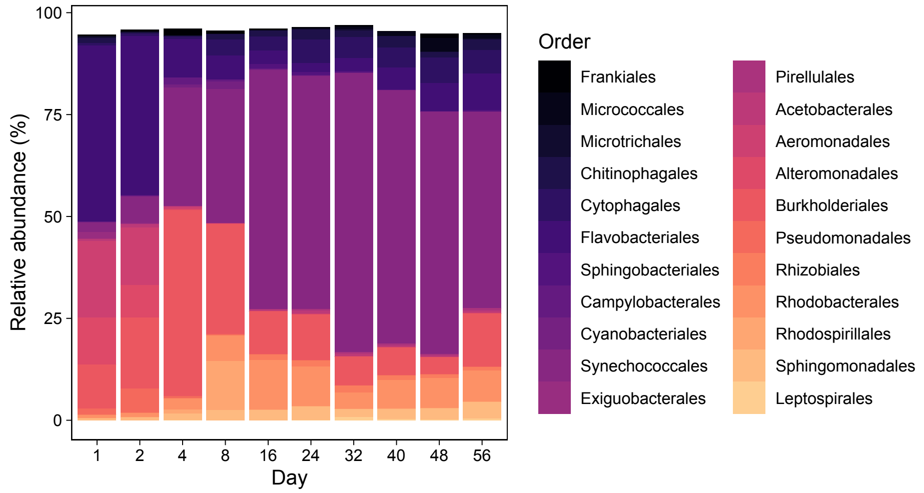

C) Large mesocosm

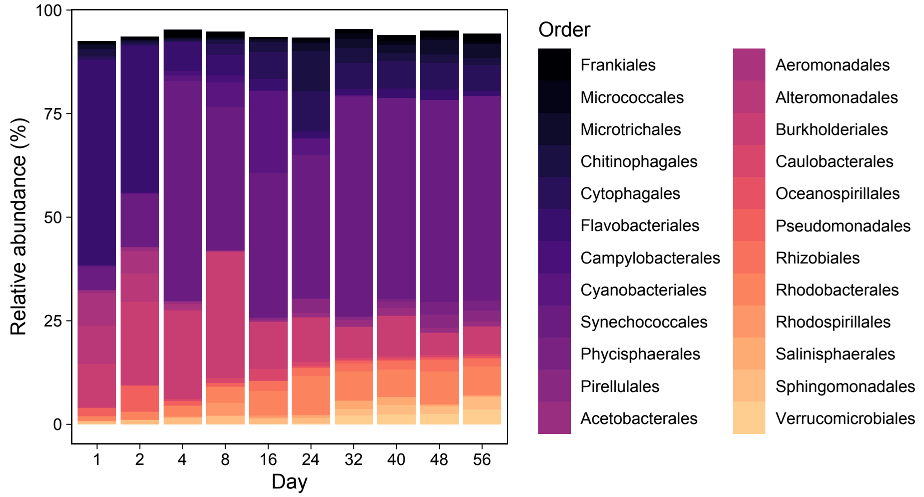

Figure S6: Relative abundance of identified taxonomic orders in small (A), medium (B), and large (B) sized mesocosms of the top 50 most abundant ASVs from day 1 to 56 with combined salinity levels.

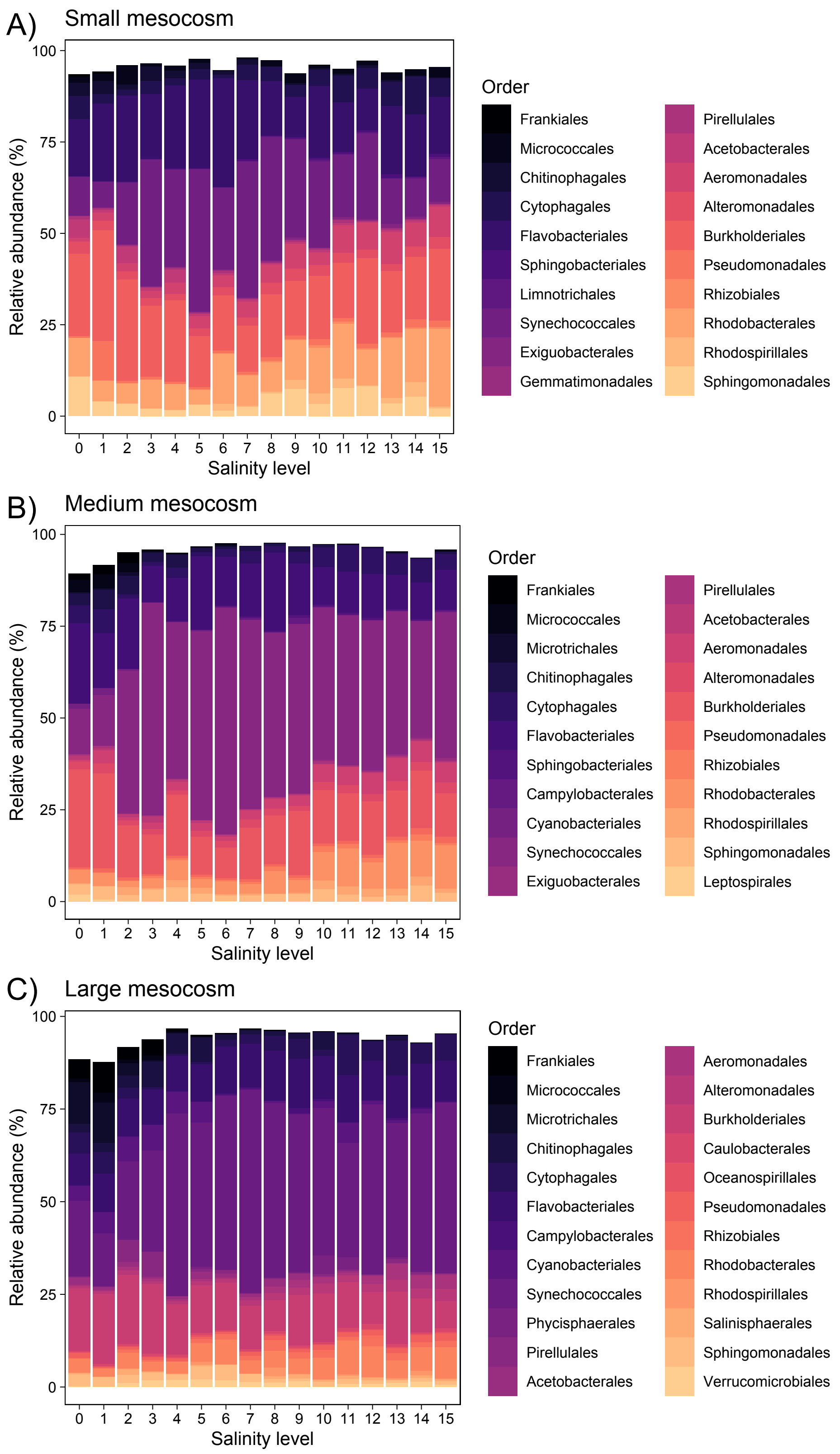

Figure S7: Relative abundance of identified taxonomic orders of the top 50 most abundant ASVs in small (A), medium (B), and large (B) sized mesocosms in each salinity level combined from day 1-56.

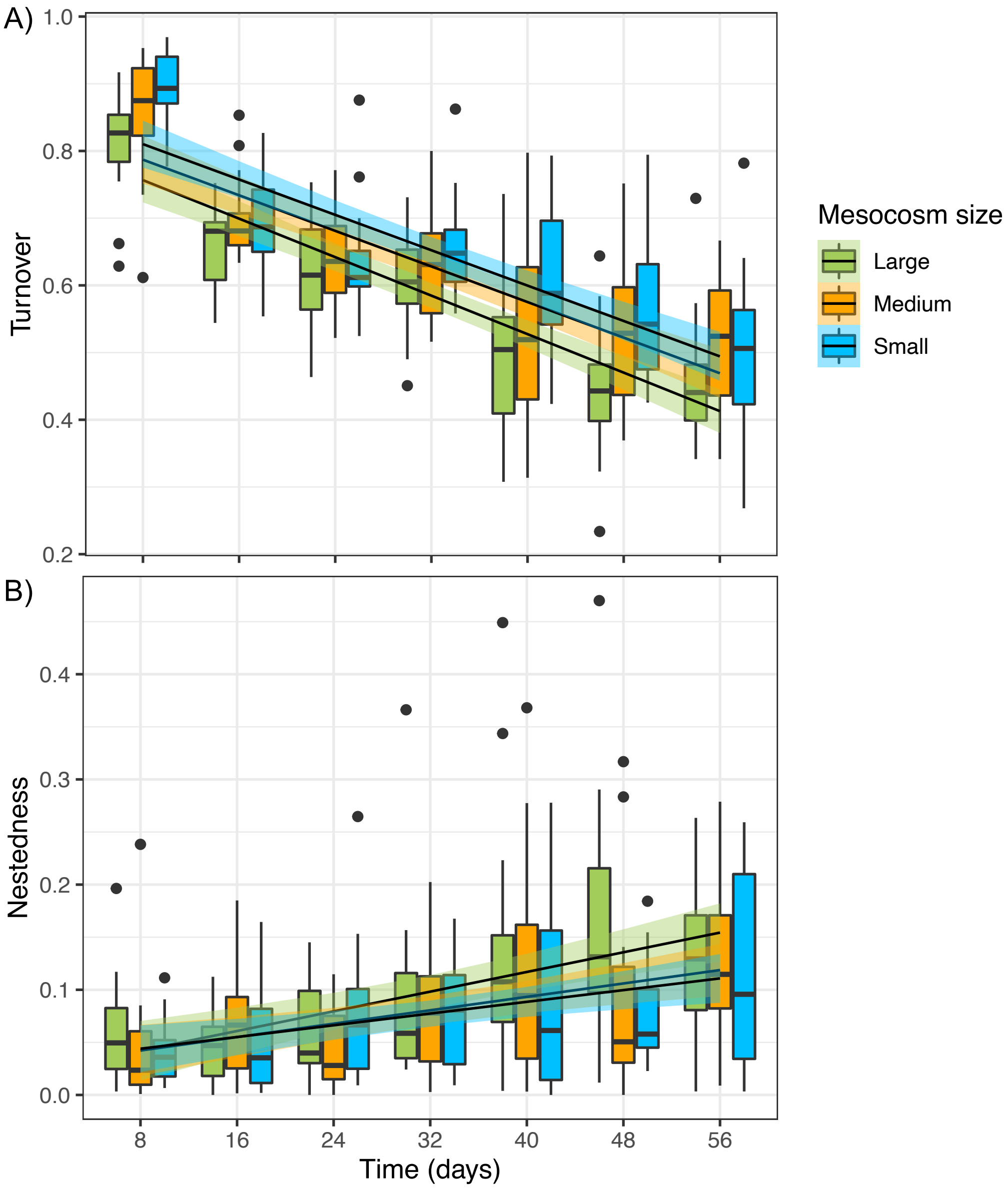

Figure S8. Beta diversity in different mesocosm sizes over time compared to the prior time point based on Jaccard dissimilarity index of taxa presence-absence and partitioned into taxa turnover (A) and nestedness (B). (n = 16)

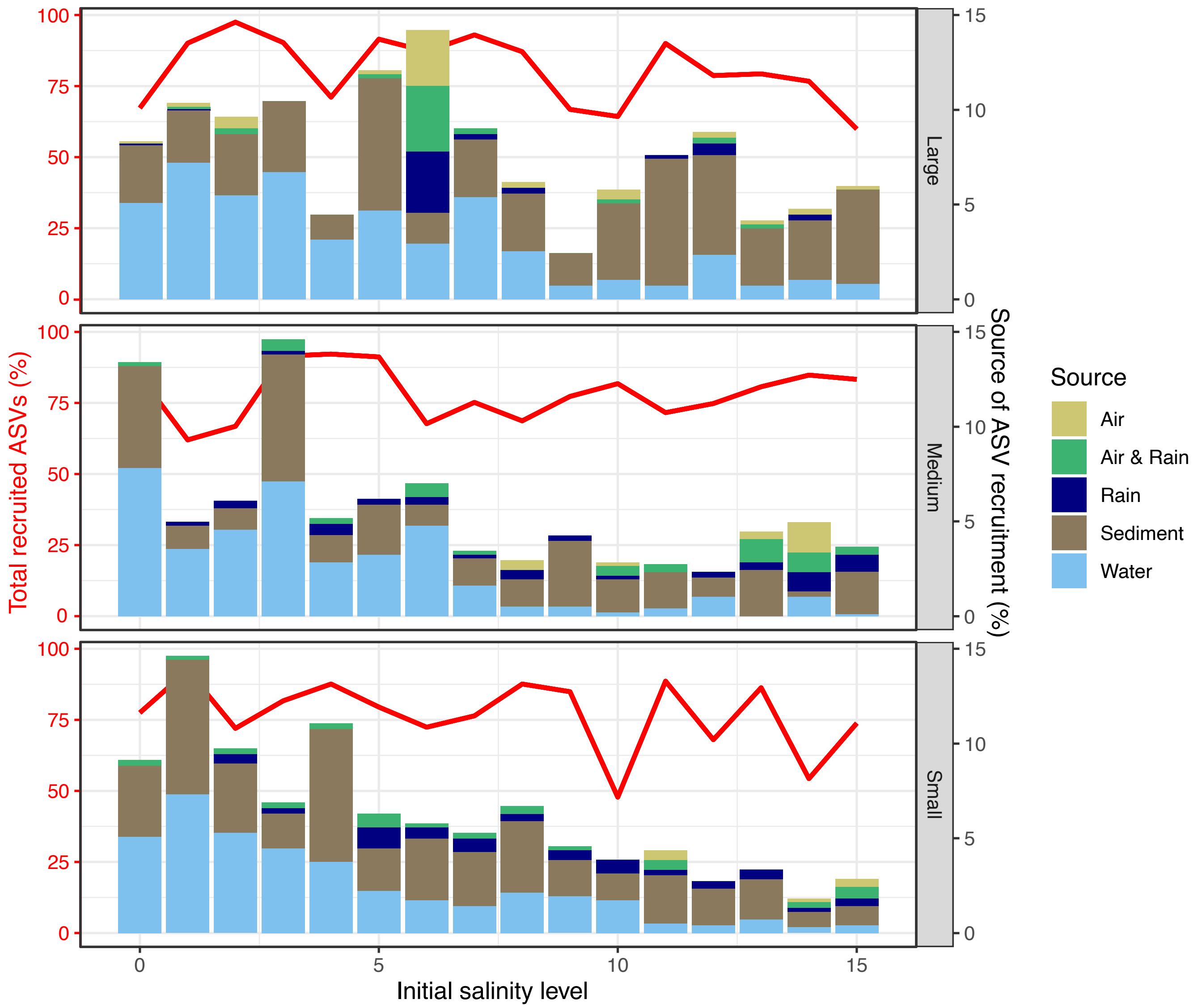

Figure S9. The percent of unique ASVs recruited over the duration of the experiment in each mesocosm at different levels of initial salinity disturbance with level 0 representing 0 ‰ salinity and level 15 representing 6 ‰ salinity. Panels are ordered by mesocosm size with large mesocosms in the top panel. The line plot represents total unique ASV recruitment including from unknown sources. The bar plot represents ASVs recruited from identified sources with the total height of the bar showing the percentage of ASVs recruited to that mesocosm from an identified source. Air and rain had 156 ASVs recruited in common.

Table S4: Standardized estimates for the path analysis of the small mesocosms. Significant effects are with a Benjamini-Hochberg correction. Standardized Root Mean Square Residual = 0.229.

**Small mesocosms**								
Environmental and community variables		Cond	Temp	Chl-*a*	CDOM	TOC	TN	TP
β_{bc} ← <J>	-0.173***							
β_{bc} ← ΔS	0.028							
β_{bc} ← Δt	0.326***							
β_{bc} ← Δx	0.076***							
β_{bc} ← ΔE	0.925‡	0.320***	0.046*	0.226***	-0.056	-0.122***	0.140***	0.015
ΔS← ΔJ	0.016							
ΔE← Δt	1.201‡	-0.022	0.558***	0.160***	0.312***	0.014	0.096**	0.039
ΔE← Δx		0.052*						

Cond = conductivity, Temp = temperature, Chl-*a* = chlorophyll-*a*
* p < 0.05 to 0.01, ** p 0.01 to 0.001, *** p <0.001
‡ Total of the absolute values of standardized estimates in the row

Table S5: Standardized estimates for the path analysis of the medium mesocosms. Significant effects are with a Benjamini-Hochberg correction. Standardized Root Mean Square Residual = 0.127.

**Medium mesocosms**								
Environmental and community variables		Cond	Temp	Chl-*a*	CDOM	TOC	TN	TP
β_{bc} ← <J>	-0.133*							
β_{bc} ← ΔS	0.084*							
β_{bc} ← Δt	0.273***							
β_{bc} ← Δx	0.013							
β_{bc} ← ΔE	0.773‡	0.511***	-0.016	-0.072	0.083	-0.009	0.057	0.025
ΔS← ΔJ	0.227***							
ΔE← Δt	1.477‡	-0.026	0.551***	0.218***	0.284***	0.235***	-0.014	0.149***
ΔE← Δx		0.283***						

Cond = conductivity, Temp = temperature, Chl-*a* = chlorophyll-*a*
* p < 0.05 to 0.01, ** p 0.01 to 0.001, *** p <0.001
‡ Total of the absolute values of standardized estimates in the row

Table S6: Standardized estimates for the path analysis of the large mesocosms. Significant effects are with a Benjamini-Hochberg correction. Standardized Root Mean Square Residual = 0.116.

Large mesocosms								
Environmental and community variables		Cond	Temp	Chl-*a*	CDOM	TOC	TN	TP
$\beta_{bc} \leftarrow \langle J \rangle$	-0.136*							
$\beta_{bc} \leftarrow \Delta S$	0.174***							
$\beta_{bc} \leftarrow \Delta t$	0.370***							
$\beta_{bc} \leftarrow \Delta x$	-0.058*							
$\beta_{bc} \leftarrow \Delta E$	0.766‡	0.427***	-0.084***	0.099*	0.075	-0.038	-0.007	-0.036
$\Delta S \leftarrow \Delta J$	0.147***							
$\Delta E \leftarrow \Delta t$	1.579‡	-0.014	0.485***	0.124***	0.349***	0.308***	0.099***	0.200**
$\Delta E \leftarrow \Delta x$		0.298***						

Cond = conductivity, Temp = temperature, Chl-*a* = chlorophyll-*a*
* $p < 0.05$ to 0.01, ** p 0.01 to 0.001, *** $p < 0.001$
‡ Total of the absolute values of standardized estimates in the row

Table S7: Summary statistics for the constructed complete (Bacteria + Env) and bacterial subnetworks of 50 most abundant Bacteria (ASVs) and environmental parameters of small, medium, and large mesocosms (Figure 3). Note that only the results of the significant associations are listed.

	**Small**		**Medium**		**Large**	
**Parameters**	**Bacteria**	**Bacteria + Env**	**Bacteria**	**Bacteria + Env**	**Bacteria**	**Bacteria + Env**
Nodes (without edge to environmental variable)	37	43 (18)	49	57 (5)	50	58 (8)
Edges	44	73	240	343	288	396
Positive-delayed edges (% of all edges)	5 (11.4%)	5 (6.8%)	33 (13.8%)	44 (12.8%)	28 (9.7%)	42 (10.6%)
Negative-delayed edges (% of all edges)	4 (9.1%)	10 (13.7%)	32 (13.3%)	60 (17.5%)	37 (12.8%)	80 (20.2%)
Diameter (radius)	7 (4)	6 (3)	6 (4)	5 (3)	8 (4)	8 (4)
Delayed/non-delayed associations	9 / 35	15 / 58	65 / 175	104 / 239	65 / 223	122 / 274
Connectivity						
Average number of neighbors	3.08	4.21	10	12.25	11.755	13.89
Network density	0.134	0.156	0.213	0.223	0.245	0.248
Likelihood for uneven distribution of edges						
Network heterogeneity	0.537	0.635	0.623	0.535	0.529	0.504
Network centralization	0.138	0.271	0.289	0.259	0.223	0.206
Identifying small-world properties						
Characteristic path length	3.014	2.643	2.435	2.17	2.69	2.615
Clustering coefficient	0.353	0.451	0.586	0.60	0.528	0.525

Env = environmental parameter
